# Brusatol Derivative–34 Attenuates Allergic Airway Inflammation Via Inhibition of the Spleen Tyrosine Kinase Pathway

**DOI:** 10.3389/fphar.2021.587417

**Published:** 2021-03-30

**Authors:** Yasi Ding, Weibin Tang, Fei Pei, Lixia Fu, Pei Ma, Jinye Bai, Mingbao Lin, Yunbao Liu, Qi Hou

**Affiliations:** ^1^ State Key Laboratory of Bioactive Substance and Function of Natural Medicines, Chinese Academy of Medical Sciences and Peking Union Medical College, Institute of Materia Medica, Beijing, China; ^2^ National Medical Products Administration, Center for Drug Evaluation, Beijing, China

**Keywords:** Bru-34, allergic airway inflammation, spleen tyrosine kinase, cytoplasmic phospholipase A2, nuclear factor-κB

## Abstract

Brusatol derivative-34 (Bru-34), a derivative of brusatol, has been shown significantly anti-inflammatory activity in mice in our previously work. However, to our knowledge, there were very limited studies on how Bru-34 affected airway inflammation. Thus, in this present study, the effects and potential mechanisms of Bru-34 on allergic airway inflammation were examined both *in vivo* and *in vitro*. The results showed that Bru-34 attenuated the allergic airway inflammation in mice, with significant decreasing of the inflammatory cells and mediators in bronchoalveolar lavage fluids and attenuation of the histopathological alterations in the lung tissues. In addition, Bru-34 significantly inhibited the release of inflammatory cytokines in antigen induced rat basophilic leukemia -2H3 (RBL-2H3) cells. What’s more, Bru-34 significantly decreased the expression of spleen tyrosine kinase (Syk), *p*-Syk, cytoplasmic phospholipase A2 (cPLA2), *p*-cPLA2, nuclear factor-κB (NF-κB) and p-NF-κB both in allergic mice lung tissue and antigen induced RBL-2H3 cells. Furthermore, the collaborative effects of Bru-34 with inhibitors against Syk, cPLA2, and NF-κB, showed that Syk was an important target of Bru-34, and cPLA2 and NF-κB played important roles in the coordinated inflammatory response. In conclusion, Bru-34 could significantly modulate the allergic airway inflammation, and its potential mechanism was revealed at least partially via down-regulating of Syk-cPLA2 -NF-κB signaling.

## Introduction

As one of the most common chronic respiratory diseases in the world, allergic asthma have affected 1–18% of the world’s population, and by 2025, it would rise to affect approximately 400 million people worldwide (Boulet et al., 2015; McIvor, 2015; Reddel and Levy, 2015). At present, inhaling glucocorticoids, long-acting bronchodilators and anti-histamines are still the mainstream therapeutic strategies for asthma (Gao et al., 2012). Although corticosteroids are the most successful anti-inflammatory medications available with only about 10% not responding (Hanania et al., 1995). Clinical studies have shown that corticosteroids significantly reduce airway inflammation and hyperresponsiveness, however, corticosteroids can cause both local and systemic side-effects, such as oropharyngeal candidiasis, dysphonia, reflex cough. Therefore, more effective with less side effect anti-asthmatic drugs are poorly needed (Kelly and Nelson, 2003).

**FIGURE 1:**
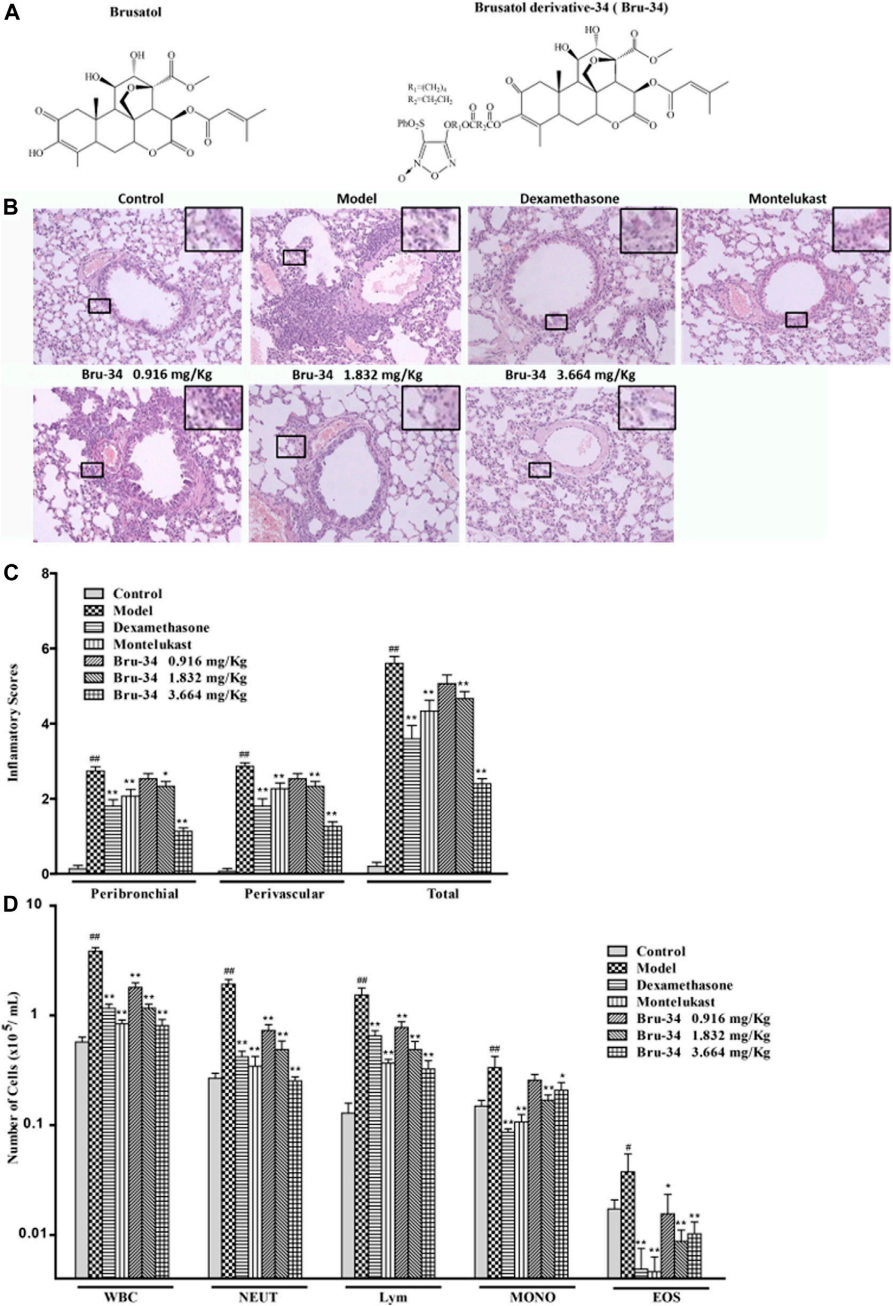
Bru-34 attenuated the inflammatory cells infiltration in OVA - dependent airway inflammation. Panel **(A)**: Chemical structures of brusatol and Bru-34; Panel **(B)**: Lung sections with hematoxylin-eosin (H&E) staining (×100); Panel **(C)**: A four-point inflammatory scores evaluated on the accumulation of inflammatory cells in H&E stained lung sections (*n* = 4); Panel **(D)**: The number of inflammatory cell infiltrated in BALF (*n* = 8); Data were expressed as mean ± S.D.; #*p* < 0.05 and ##*p* < 0.01 vs. control group by Student’s *t*-test statistical analysis; *p < 0.05 and **p < 0.01 vs. model group by one-way ANOVA statistical analysis, the same to the following figures.

In recently years, natural products and their derivatives have shown anti-inflammation potential for chronic respiratory diseases, such as asthma and chronic obstruct pulmonary disease (COPD). Brusatol (Figure 1A), a nature product isolated from the medicinal plants belonging to Brucea genus, has been demonstrated to show a variety of pharmacological activities, including anti-tumor, anti-malaria, anti-inflammatory, and anti-viral activities (Tang et al., 2014). However, its high toxicity severely limited its clinical application. Therefore, in order to improve its anti-inflammatory activity and reduce the toxicity, in our previous studies, brusatol derivatives were designed and synthesized based on the new strategy of the conjugation of brusatol with NO-donor furoxan substructures, which endowed the new compounds with various NO-releasing capabilities (Tang et al., 2014). Among them, brusatol derivative - 34 (Bru-34, Figure 1A) exhibited significant anti-inflammatory activities both *in vivo* and *in vitro*, with much less toxicity compared to brusatol (Tang et al., 2014). However, the potential effect of Bru-34 on the allergic airway inflammation and its underlying mechanism are still unavailable.

Allergic asthma is characterized by airway eosinophilia, airway obstruction and bronchial hyperresponsiveness associated with a T helper 2 (Th_2_) lymphocyte immune response to environmental allergens (Vanderstocken et al., 2010). Its key mechanism is characterized by hyper-production of allergen-specific IgE responding to antigens, which subsequently binds with high affinity Fc receptors (FcεRⅠ) on the surface of mast cells and basophils, and consequently caused their degranulation and release of biologically active molecules, which mediates airway allergic inflammation (Freidhoff and Marsh, 1993; Gould and Sutton, 2008; Cheng et al., 2013).

The Syk plays a critical role in FcεRⅠ-dependent acute inflammation (Oliver et al., 2000). Syk expression increased significantly in the airway epithelia of chronic mouse model of asthma. A lot of research have demonstrate that inhibiting SYK blocks mast cell degranulation and attenuates airway hyperresponsiveness (Penton et al., 2013; Wong and Leong, 2004). It is recruited and activated by the phosphorylation of immunoreceptor tyrosine-based activation motifs (ITAMs) of the FcεRI complex, which is mediated by the cross-linking allergen with FcεRⅠ (Gilfillan and Rivera, 2009). By activating multiple downstream signaling processes including NF-κB, phospholipase Cγ1 (PLCγ1) and PLA_2_, etal,Syk could cause the activation of several enzymes, adaptors and other related biochemical molecules, which leading to the secretion of inflammatory mediators and pro-inflammatory cytokines, production of the arachidonic acid metabolites, and degranulation in mast cells and basophils (Ulanova et al., 2005; Lu et al., 2014). Hence, Syk is considered as a useful new therapeutic target for allergic inflammation.

In the present study, we examined the effects of Bru-34 on allergic airway inflammation and investigated the roles of Syk and its downstream signaling molecules (NF-κB and cPLA_2_) in the Bru-34’s anti-inflammation procession.

## Materials and Methods

### Materials

All of the antibodies against the indicated proteins and other reagent used in this study are listed as follows: anti-NF-κB mouse antibody (Cat#, 8242s), anti-Phospho-NF-κB antibody (Cat#, 3033s),anti-Phospho-Syk antibody (Cat#, 2,710), anti-cPLA 2 antibody (Cat#, 2,832), anti-Phospho- cPLA 2 antibody (Cat#, 2,831),from Cell Signal Technology; anti-Syk Rabbit antibody (Cat#, ab3782) from Abcam;β-actin rabbit antibody (Cat#, p30002) from Abmart; Western Blot ECL Kit (Cat#, 34,095) was purchased from Thermo Scientific;Syk kinase Enzyme System (Cat#, V3801), ADP-Glo™ kinase Assay (Cat#, V9101) was purchased from Promega; Rat TNF-α ELISA Kit (Cat#, 438,204) was purchased from Biolegend; Rat IL-4 ELISA Kit (Cat#, DY504) was purchased from R&D; Ovalbumin (Cat#, 080M7012V) was purchased from Sigma.

### Animal Care

The experimental protocol was approved by the Ethics Committee for Animal Experimentation of Institute of Materia Medica, Chinese Academy of Medical Sciences & Peking Union Medical and conformed to internationally accepted ethical standards. Balb/c mice (males, 16–18 g weights) were supplied by the Animal Center of Military Medical Science (Beijing, China) and housed under controlled conditions with a 12 h light/dark cycle, at a temperature of 21 ± 2 C, and humidity of 60 ± 5% for 1 week before experimentation. The animals were allowed free access to a standard rodent diet and tap water.

### Ovalbumin-Induced Allergic Airway Inflammation and Treatment

Mice were sensitized with intra peritoneal injection (i.p.) of OVA 20 μg plus alum hydroxide (Meihua Chemical Industry Limited Company of Shanghai, China) 1 mg in 0.2 ml saline on days 0, 7 and 14, and challenged by intratracheal instillation with intranasal ovalbumin 80 μg in 50 μL saline on days 26–28, then harvested on day 29. The control animals were sensitized and challenged with normal saline in same volume.

On days 15–28, mice were intragastric administrated with normal saline (control group), Bru-34 0.916, 1.832, 3.664 mg/kg (1, 2, 4 μmol/ kg; synthesized by the Institute of Materia Medica of the Chinese Academy of Medical Sciences, Beijing, China), montelukast Sodium (MT) 1.52 mg/kg (2.5 μmol/kg; Merck Sharp & Donhme Ltd., United Kingdom) and dexamethasone sodium phosphate (DEX) 0.516 mg/kg (1 μmol/kg; China National Medicines Corporation Ltd., Beijing, China).

### Quantification of Inflammatory Cells in the Bronchoalveolar Lavage Fluids

Twenty-four hours after the last challenge, mice were anesthetized and bled by retro-orbital puncture with capillary tubes for collecting of blood samples, and then BALF was collected by intratracheal instillation of 700 μL of PBS triply. The BALF was centrifuged and the supernatants were stored at −80 C for ELISA assays. Cell pellets were resuspended in 0.5 ml of PBS, and total cell numbers, neutrophils, lymphocyte, monocyte and eosnophils in BALF were counted in a hematology counter (Beckman Coulter LH 750, United States).

### Analysis of Cytokines in Bronchoalveolar Lavage Fluids and Serum

The concentrations of multiple cytokines (TNF-α, IL-1β, IL-6, IL-17A, IL-4 and IFN-γ) in BALF and the levels of ovalbumin-specific IgE and IgG1 in serum were determined by using ELISA kits, according to their responding protocols.

### Histological Examination and Analysis

Lung tissues were fixed in 10% neutral buffered formalin solution for 24 h, and then paraffin embedded, and the sections (4 μm) were stained with H&E according to standard protocols. The morphometric analysis of inflammatory cell infiltration was performed under light microscopy (100×), selecting venules with diameter of 100–200 μm and bronchi with 150–300 μm of diameter, five blood vessels or bronchi from four lung sections for each experimental group. The results were expressed as average on a four-point scale (Fan et al., 2019). The observer who performed the evaluation was blind to the group.

### Immunohistochemistry Analysis

Immunohistochemistry was performed as previously described (Xuan et al., 2014). The 4 μm Paraffin-embedded tissue sections were deparaffinized in xylene, rehydrated through graded alcohols, and rinsed in PBS. Endogenous peroxidase activity was blocked with 3% H_2_O_2_, and then non-specific binding was blocked with 10% normal goat serum. The slides were subsequently incubated with a rabbit anti-p-NF-κB antibody at 4 C for overnight, and then incubated with goat anti-rabbit second antibody. The sections were stained with an ABC kit (Zhong Shan Golden Bridge Biotechnology Co., Ltd., Beijing, China), and dehydrated through graded alcohol and mounted with neutral gum. The sections were observed under a microscope, and the results were expressed as the mean integrated optical density of immunostaining, performed using Image-Pro Plus 6.0 software.

### Cell Culture and Treatment

RBL-2H3 cells (Type Culture Collection of the Chinese Academy of Sciences, Shanghai, China) were cultured in Dulbecco’s modified Eagle’s medium (4,500 mg/L glucose) supplemented with 10% (v/v) fetal bovine serum (Gibco) at 37 C in a humidified atmosphere of 5% (v/v) CO_2_. Cells were seeded at 1 × 10^5^ cells per well on 48 well plates. After 12 h, cells were sensitized with anti-DNP IgE (1 μg/ml) for 12 h. Before stimulated with DNP-BSA (1 μg/ml) for 12 h (cytokines Elisa test), cells were pretreated with different concentrations of test compounds for 1 h, and the control cells were cultured under normal conditions with vehicle. After stimulation, the cell culture supernatants were collected for cytokines detection by ELISA. The manufacturer’s instructions were strictly followed during the ELISA experiments.

### Measurement of *β*-hexosaminidase Release

After pretreated with different concentrations of test compounds for 12 h, the cells were stimulated with DNP-BSA (1 μg/ml) in PIPES buffer for 30 min, and the control cells were cultured under normal conditions with vehicle. After DNP-BSA stimulation, 50 μL of cell supernatant and 50 μL of 1 mM *p*-nitrophenyl-N-acetyl-*β*-Dglucosaminide in 100 mM citrate buffer (pH 4.5) were incubated for 1 h at 37 C, and 200 μL of carbonate buffer (0.1 mM NaHCO_3_/Na_2_CO_3_, pH 10.0) was added to stop the reaction. Then, the absorbance was measured at 405 nm using a microplate reader (Power Wave XS2, BioTek, United States). The *β*-Hex release (%) was calculated: *β*-Hex release (%) = 100 - (A - T)/A × 100. A represents the average OD value of the model group, T represents the OD value of each test well.

### Western Blotting Analysis

Protein was extracted from the lung tissues and cells according to a previously described procedures (Lin et al., 2015). Membranes were probed with primary antibodies against Syk, *p*-Syk (Tyr525/526), cPLA2, *p*-cPLA2, and NF-κB (1:1,000). Horseradish peroxidase-conjugated anti-rabbit or anti-mouse secondary antibodies were used and visualized using the enhanced chemiluminescence kit. The density of each band was quantified with ImageJ 1.42q software (National Institutes of Health, United States). *β*-actin (1:5,000, Abmart, China) was used as a loading control.

### *In Vitro* Syk Kinase Assay

The experiments were carried out according to manufacturer’s instructions of Syk kinase Enzyme Systemand ADP-Glo™ kinase Assay. The Syk kinase (1 ng/μL) 2 μL, subsrate (Poly E4Y1 0.2 μg/μL)/ATP (10 μM) mix 2 μL, and different concentrations of Bru-34 (10^−4^, 10^−5^, 10^−6^, 10^−7^, 10^−8^, 10^−9^ M) or DMSO 1 μL were added to the wells of 384 low volume plate, and incubated at room temperature for 30 min. After that, 5 μL ADP-Glo™ reagent were added to each well and incubated at room temperature for 40 min. Then, 10 μL kinase detection reagent were added and incubated at room temperature for another 30 min. Luminescence signal was recorded with a multi-label plate readers (EnSpire Alpha, PerkinEImer, United States).

### Statistical Analysis

Statistical analyses were performed with PASW Statistics 18.0 software (IBM Inc.) and statistical significance was set at *p* < 0.05 or *p* < 0.01. Data was presented as the mean ± SD. As the normality test by Kolmogorov-Smirnov test (K-S test) was passed, data was analyzed by using the Student’s t-test for comparison between two groups and one-way analysis of variance (one-way ANOVA) for multiple groups followed by Fisher’s least significant difference (LSD) test, otherwise, by using Kruskal-Wallis H test.

## Results

### Brusatol Derivative-34 Attenuated the Infiltration of Inflammatory Cells in Allergic Airway Inflammation Mice

The effect of Bru-34 on airway inflammation of the lung sections was assessed by H&E-staining (Figure 1B). The quantification of peribronchiolar and perivascular inflammatory cell accumulation were determined using an inflammatory score (Figure 1C). As expected, lung histology revealed that the mice sensitized and challenged with OVA (model mice) were induced predominantly inflammatory cell infiltration in the peribronchial and perivascular regions compared to the control mice (without OVA sensitization and challenge).Compared to the model mice, the accumulation of inflammatory cells in the lung tissues was significantly reduced in Bru-34 treated mice (*p* < 0.05 or *p* < 0.01).

As shown in Figure 1D, in agreement with the histologic appearance of the lung tissues, Bru-34 treatment attenuated the levels of inflammatory cells infiltration in BALF compared to the control mice. Bru-34 treatment significantly reduced the total amount of BALF immune cells, and significantly attenuated the infiltration of eosinophils, macrophages, lymphocytes, and neutrophils in the BALF leukocyte populations (*p* < 0.05 or *p* < 0.01). Furthermore, the numbers of total cells, neutrophils and lymphocytes were reduced in a dose-dependent manner.

### Brusatol Derivative-34 Treatment Decreased the Levels of IgE and IgG1 in Serum of Allergic Airway Inflammatory Mice

**FIGURE 2:**
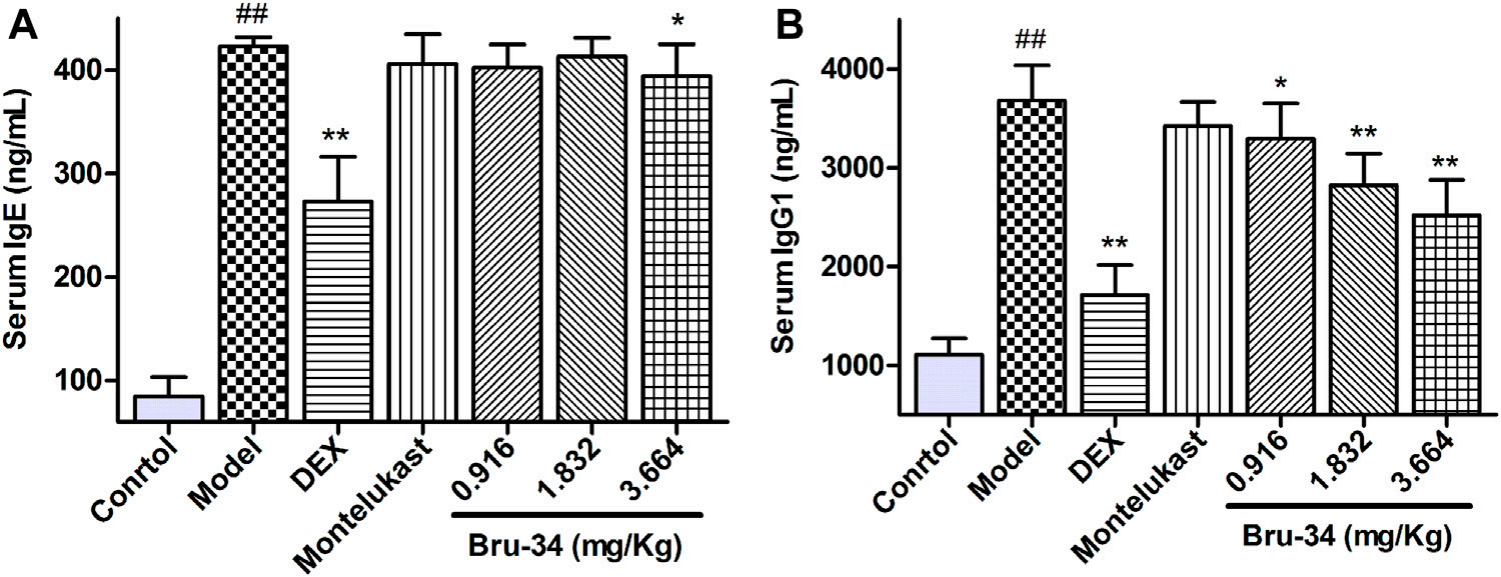
Bru-34 decreased the levels of IgE and IgG1 in serum. Panel **(A)**: The level of serum IgE; Panel **(B)**: The level of serum IgG1; Data were expressed as mean ± S.D., *n* = 8.

The Levels of IgE and IgG1 in serum were determined by ELISA. Compared to the control mice, mice sensitized and challenged with OVA revealed a significant increase in OVA-specific IgE and IgG1 production in serum. Compared to the model mice, the IgE level was significantly decreased in 3.664 mg/kg Bru-34 treatment mice (Figure 2A), and the levels of IgG1 in all treatment mice (Figure 2B).

### Brusatol Derivative-34 Treatment Decreased the Concentrations of Inflammatory Mediators in Bronchoalveolar Lavage Fluids of Allergic Airway Inflammatory Mice

**FIGURE 3:**
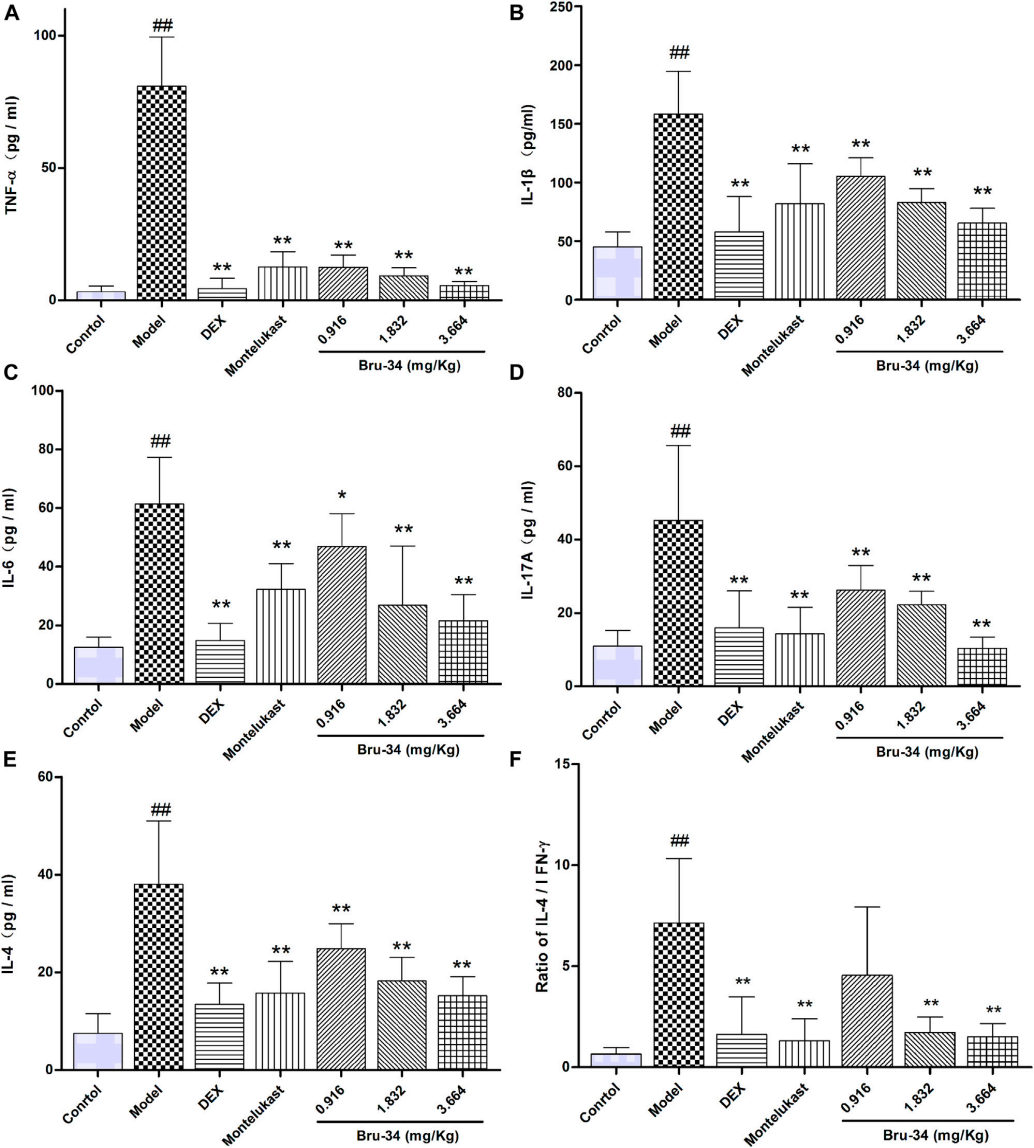
The concentrations of inflammatory mediators in BALF were determined by ELISA. Panel **(A)**: TNF-α; Panel **(B)**: IL-1β; Panel **(C)**: IL-6; Panel **(D)**: IL-17A; Panel **(E)**: IL-4; Panel **(F)**: The ratios of IL-4/IFN-γ; Data were given as mean ± S.D., *n* = 8.

The level of inflammatory mediators were determined by ELISA. Compared to model mice, Bru-34 treatment significantly attenuated the secretion of multiple cytokines in BALF, including TNF-α (Figure 3A), IL-1β (Figure 3B), IL-6 (Figure 3C), IL-17A (Figure 3D) and IL-4 (Figure 3E) in a dose-dependent manner (*p* < 0.05 or *p* < 0.01). Importantly, Bru-34 treatment significantly decreased the IL-4 level, but had no effect on the concentration of IFN-γ (Data not shown), resulting a significantly restoration of IL-4/IFN-γ ratio imbalance (Figure 3F), which shown to mediate Th2 allergic airway inflammatory phenotypes. In addition, Bru-34 treatment had no effect on IL-5, IL-10 and eotaxin release in BALF (data not shown). Collectively, Bru-34 treatment attenuated OVA-dependent secretion of BALF inflammatory mediators, in which a Th2 allergic inflammatory response was shown to being restored.

### Brusatol Derivative-34 Treatment Suppressed the Expression of Spleen Tyrosine Kinase, Cytosolic Phospholipase A2 and NF-κB in Allergic Airway Inflammatory Mice Lung Tissues

**FIGURE 4:**
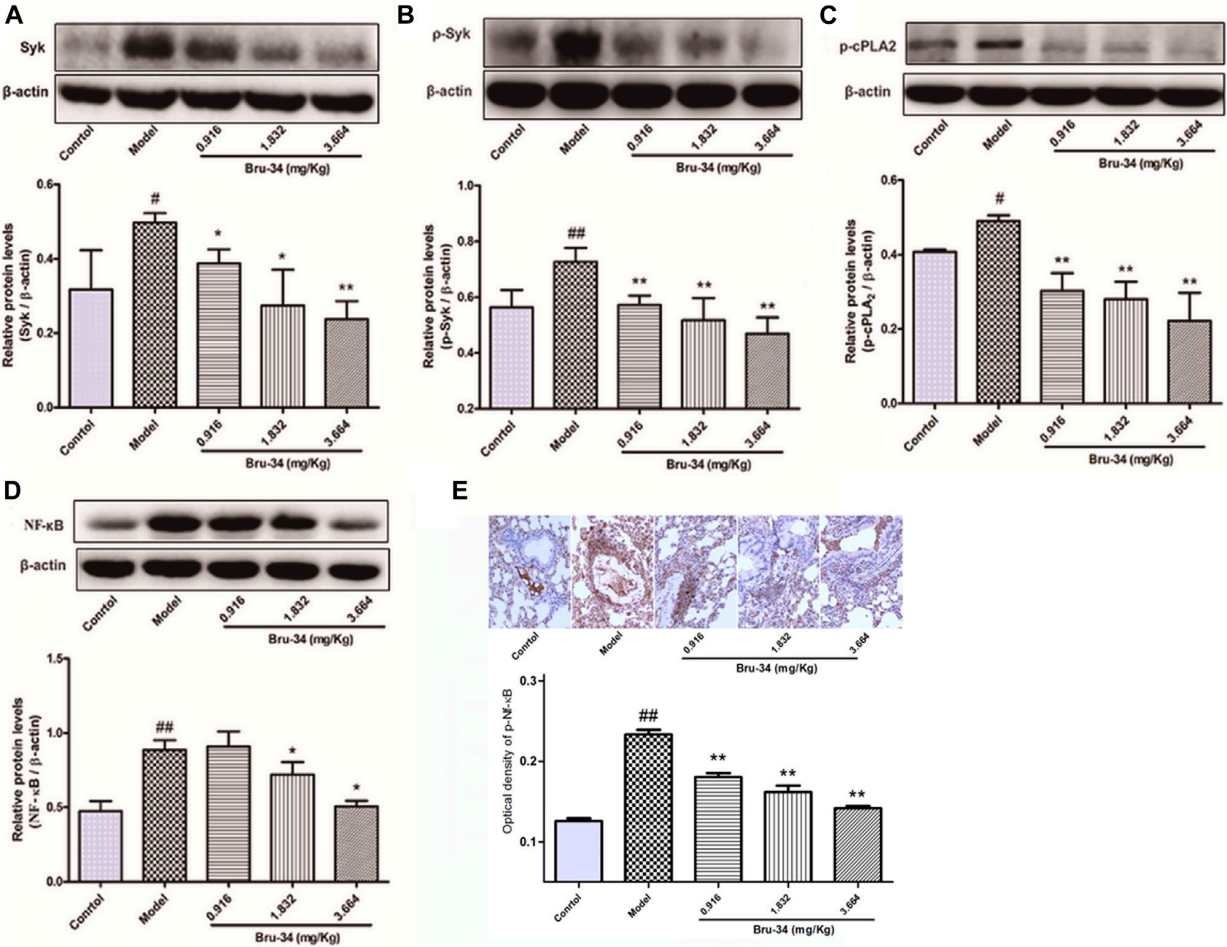
The effects of Bru-34 on Syk, cPLA2 and NF-κB expression and activation *in vivo*. Panel **(A)**: the level of Syk determined by Western blotting; Panel **(B)**: the level of *p*-Syk determined by Western blotting; Panel **(C)**: the level of *p*-cPLA2 determined by Western blotting; Panel **(D)**: the level of NF-κB determined by Western blotting; Panel **(E)**: the level of p-NF-κB were determined by immunohistochemistry - staining; Data were given as mean ± S.D. (n = 3).

Western blotting analysis was used to evaluate the effects of Bru-34 on Syk, *p*-Syk, cPLA2, *p*-cPLA2 and NF-κB Protein activation in lung tissues of allergic airway inflammatory mice, and immunohistochemistry analysis on p-NF-κB. As shown in Figure 4, compared to control, OVA-sensitized and challenged (model) mice showed a notably increase in all protein levels detected (*p* < 0.05 or *p* < 0.01). Except the cPLA2 (data not shown), Bru-34 treatment significantly attenuated the Syk (Figure 4A), *p*-Syk (Figure 4B), *p*-cPLA2 (Figure 4C), NF-κB (Figure 4D) and p-NF-κB (Figure 4E) alterations in a dose-dependent manner (*p* < 0.05 or *p* < 0.01) in comparison with the model group. Collectively, the results suggested that the potential mechanism of Bru-34 attenuating the allergic airway inflammations was via mediating the Syk pathway.

### Brusatol Derivative-34 Attenuated IgE–Antigen Induced Inflammation *In Vitro*

**FIGURE 5:**
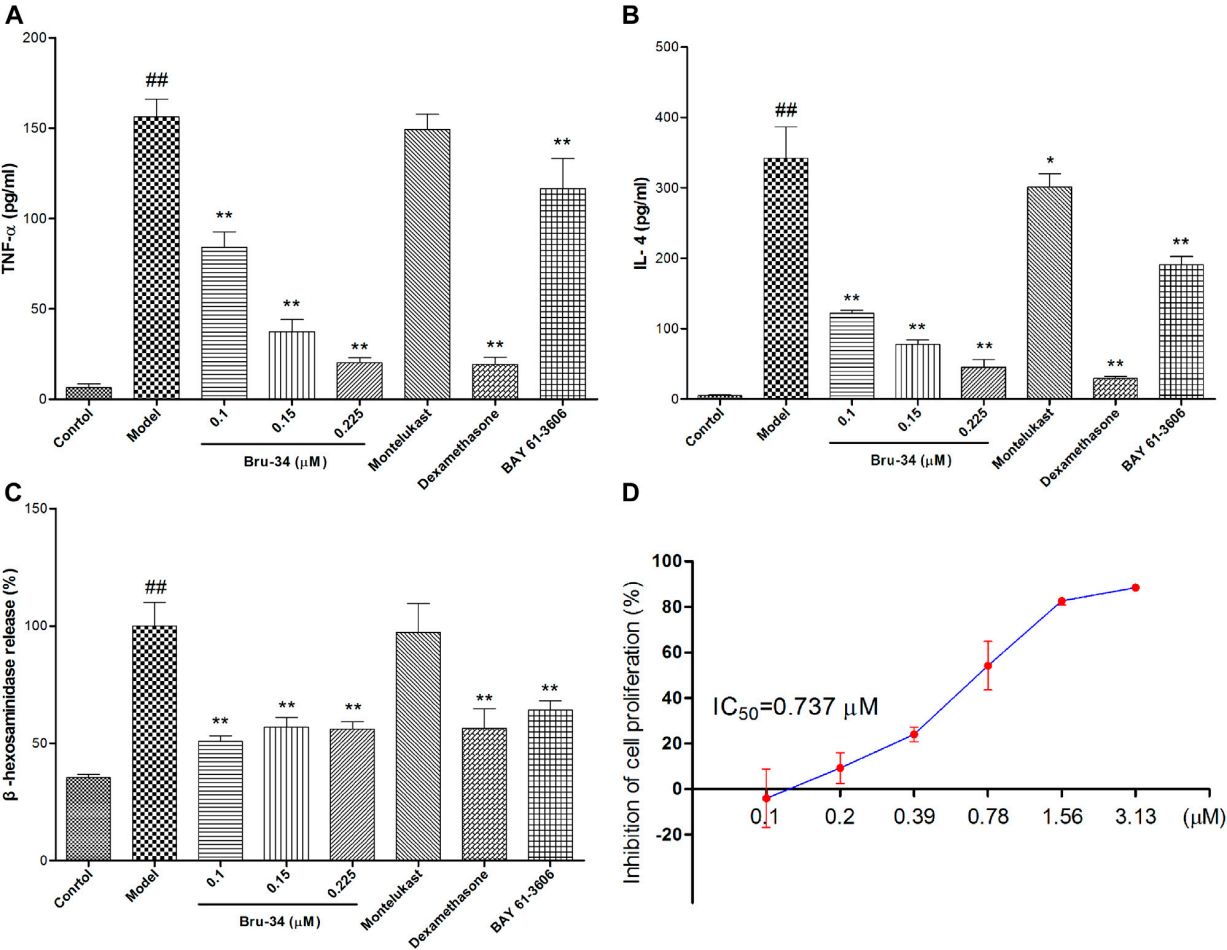
Bru-34 attenuated IgE antigen-induced inflammation in RBL-2H3 cells. Panel **(A)**: The level of TNF-α in cell culture supernatant; Panel **(B)**: The level of IL-4 in cell culture supernatant; Panel **(C)**: The release of *β*-hexosaminidase in cell culture supernatant; Panel **(D)**: The inhibition of cell proliferation; Data were given as mean ± S.D. *n* = 8.

IgE-antigen induced allergic inflammation in RBL-2H3 cells was used to evaluate anti-allergic inflammatory activity of Bru-34 *in vitro*. The release of inflammatory cytokines TNF-α (Figure 5A) and IL-4 (Figure 5B) were significantly suppressed by Bru-34 in a dose-dependent manner. Furthermore, Bru-34 markedly inhibited antigen-induced degranulation by measuring the release of *β*-hexosaminidase (Figure 5C). Additionally, Bru-34 treatment (0–0.225 μM) for 12 h produced no significant cytotoxic effect (Figure 5D).

### Brusatol Derivative-34 Inhibited Spleen Tyrosine Kinase Activity *In Vitro* and Decreased the Expression Level of Spleen Tyrosine Kinase in Antigen-Induced Rat Basophilic Leukemia-2H3 Cells

**FIGURE 6:**
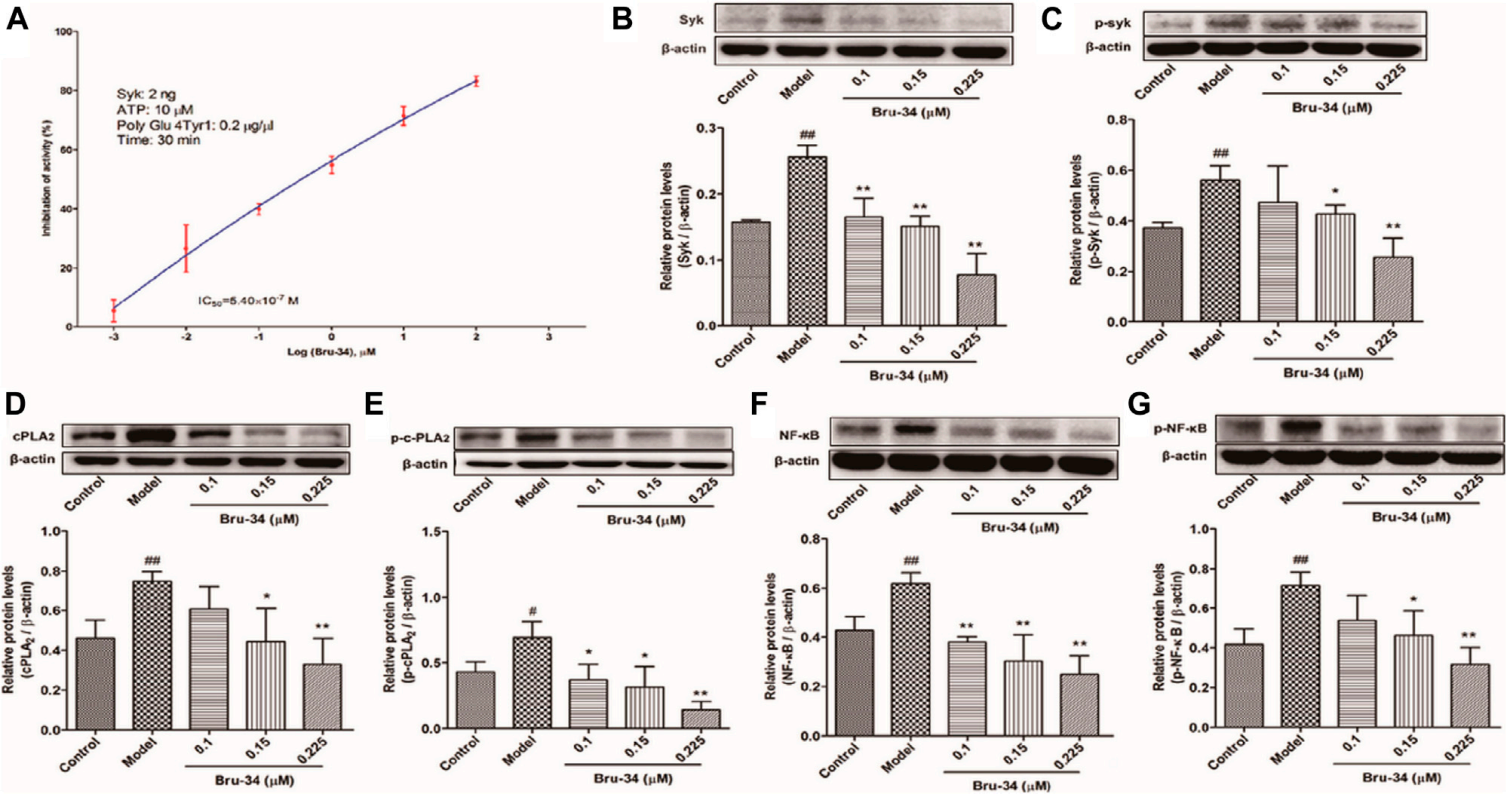
Bru-34 inhibited Syk kinase activities *in vitro* and suppressed the protein expression in antigen induced RBL-2H3 cells. Panel **(A)**: IC50 determination of Bru-34 on Syk kinase activity *in vitro*; Panel **(B)**: the level of Syk determined by Western blotting; Panel **(C)**: the level of *p*-Syk determined by Western blotting; Panel **(D)**: the level of cPLA2 determined by Western blotting; Panel **(E)**: the level of *p*-cPLA2 determined by Western blotting; Panel **(F)**: the level of NF-κB determined by Western blotting; Panel **(G)**: the level of p-NF-κB determined by Western blotting; Data were given as mean ± S.D. *n* = 3.

The mechanisms of anti-allergic inflammation of Bru-34 via mediating Syk activity were also investigated *in vitro*. Kinase assays were performed by using purified active Syk protein to confirm that Syk was the cellular target. As shown in Figure 6A, Bru-34 inhibited Syk kinase activities with an IC50 of 5.40 ×10^−7^ M. Notably, Bru-34 treatment markedly suppressed IgE antigen-induced expression of Syk (Figure 6B) and the phosphorylation of Syk (Figure 6C) in RBL-2H3 cells, showing a dose-dependent manner. Furthermore, the expression of cPLA2 (Figure 6D) and *p*-cPLA2 (Figure 6E) were inhibited with a similar potency by Bru-34. Additionally, Bru-34 dose-dependently suppressed the activation of NF-κB and p-NF-κB in IgE-activated RBL-2H3 cells (Figures 6F,G).

### Brusatol Derivative-34 Attenuated IgE-Induced Inflammation *In Vitro* Is Associated With Inhibition of the Spleen Tyrosine Kinase and Its Downstream Mediators

**FIGURE 7:**
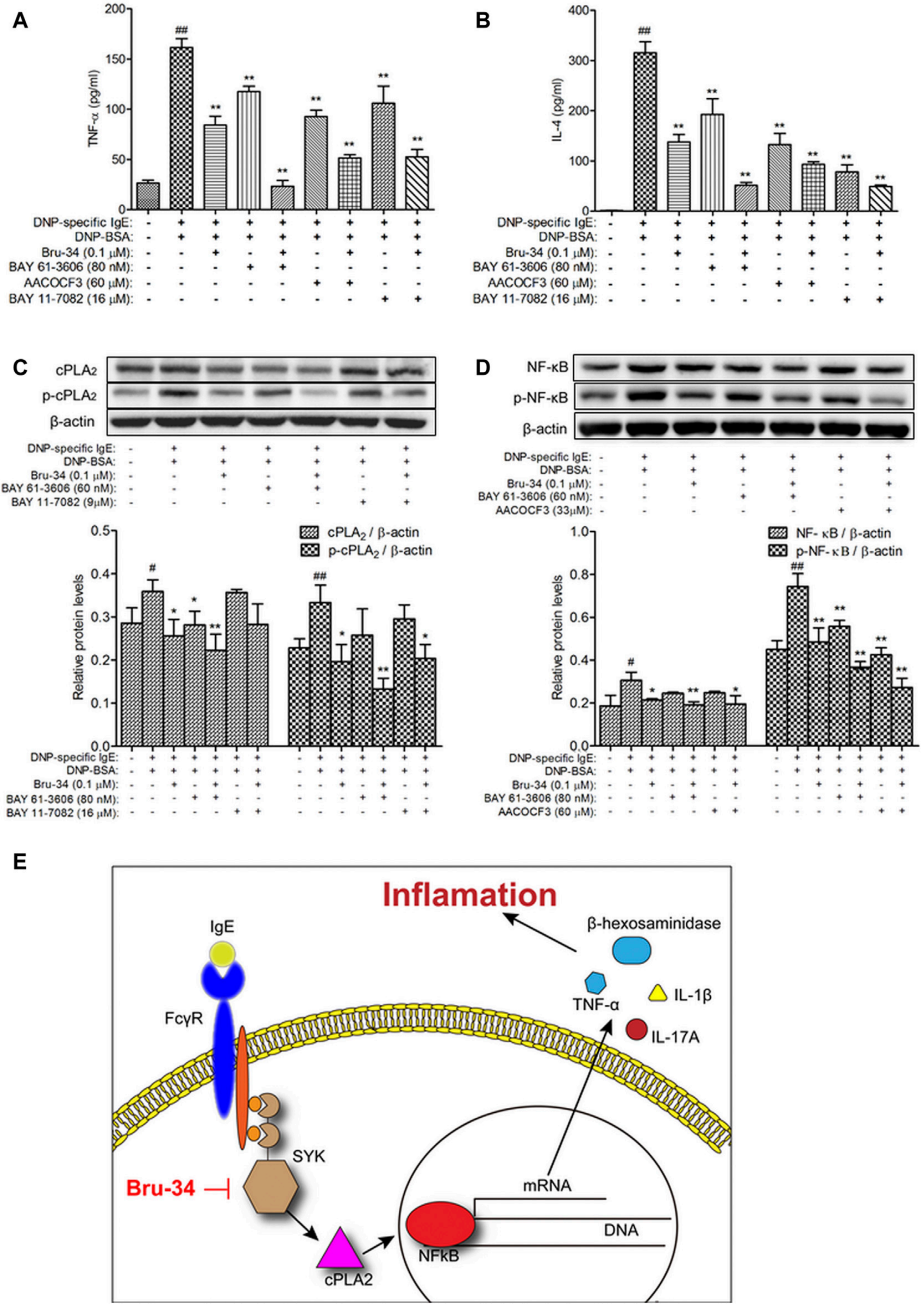
Verification that Syk, cPLA2 and NF-κB were associated with Bru-34attenuated IgE-induced Inflammation *in vitro*. Panel **(A)**: The concentrations of TNF-αin the supernatants were quantified by ELISA (*n* = 8); Panel **(B)**: The concentrations ofIL-4 in the supernatants were quantified by ELISA (*n* = 8); Panel **(C)**: Cell lysates fromSyk and NF-κB inhibitors treated cells were collected, cPLA2 and p-cPLA2 wereanalyzed by Western blotting (*n* = 3); Panel **(D)**: Cell lysates from Syk and cPLA2inhibitors treated cells were collected, NF-κB and p-NF-κB were analyzed by Westernblotting (*n* = 3); BAY 61-3606: syk inhibitor, AACOCF3: cPLA2 inhibitor, BAY11-7082: NF-κB inhibitor; Data were given as mean ± S.D.; Panel **(E)**: Schematicillustrating the mechanism by which Bru-34 Attenuates Allergic Airway Inflammationvia Inhibition of the Syk Pathway.

To examine the role of Syk in the protective effects of Bru-34 on allergic Inflammation, Syk inhibitor and its downstream cPLA2 and NF-κB inhibitors were employed. As shown in Figures 7A,B, treatments with Syk, cPLA2 and NF-κB inhibitors significantly reduced the IgE-induced TNF-α and IL-4 production, and which were almost completely abolished by that Bru-34 act collaboratively with the inhibitors. Interestingly, Syk inhibitor decreased the expression of cPLA2 and NF-κB, which were superimposed by co-treating with Bru-34 (Figures 7C,D). cPLA2 inhibitor significantly decreased the expression of p-NF-κB (Figure 7D), while, NF-κB inhibitor did not affect the protein expression of cPLA2 and *p*-cPLA2. These data collectively revealed that Syk was the important target, through which Bru -34 mediated the inflammatory response. Results also indicated that cPLA2 and NF-κB, molecules downstream of Syk, played an important role in coordinated the inflammatory response (Figure 7E).

## Discussion

Allergic asthma is one of the most common chronic respiratory diseases, Although corticosteroids can significantly reduce airway inflammation and hyperresponsiveness, however, corticosteroids can cause some side-effects. Natural products and their derivatives have recently been proved as innovative method for the treatment of chronic respiratory diseases, such as asthma. In this study, using OVA-induced allergic airway inflammation model, we observed a strong anti-allergic airway inflammatory properties of Bru-34, a derivative of brusatol, which is a nature product isolated from the medicinal plants belonging to Brucea genus. Compared with brusatol, Bru-34 is more effective with less toxicities (Tang et al., 2014).

As a chronic airway inflammatory disease, asthma is characterized by pulmonary air flow obstruction, airway mucosal inflammation, and airway hyperresponsiveness. Various inflammatory cell participates this process, such as eosinophils, macrophages, lymphocytes, and neutrophils. Quantification of this cells in the BALF could directly reflect the airway inflammation conditions. In our study, we observed an elevated number of inflammatory cells in OVA-induced model mouse, while Bru-34 treatment reduced the number of inflammatory cells in a dose dependent manner. Meanwhile, the secretion of inflammatory cytokines such as TNF-α, IL-1β, IL-6, IL-17A and IL-4 induced by OVA was also inhibited by Bru-34 treatment. Moreover, the pathological results also suggested that Bru-34 treatment led to a significant reduction in airway inflammatory cells infiltration.

Since their establishment in 1981, RBL-2H3 cells have been widely used as a mast cell model. RBL cells are usually used as tools for the study of intracellular membrane trafficking and exocytosis and the detection of allergens, vaccine safety studies and diagnosis of allergic sensitization (Passante et al., 2009; Falcone et al., 2018). After activated by IgE, mast cell could degranulate and release large amounts of inflammatory cytokines such as *β*-hexosaminidase, TNF-α, and IL-4. Consistent with *in vivo* results, Bru-34 treatment also attenuated the antigen induced inflammatory cytokines secretion in IgE-activated mast cells *in vitro*.

Lots of study have show that Th1/Th2 immune imbalance may participate in the process of asthma. Here, we demonstrated that Bru-34 markedly restored the Th1/Th2 immune imbalance in asthma by lowering the level of IL-4 and IL-4/IFN-γ ratio in BALF of allergic mice. The cytokines (IL-4, 5, 6, 10, and 13) produced by Th2 cells have been shown to play pivotal roles in the development of eosinophilic inflammation in asthma, while interleukin-2 and interferon-γ were produced by Th1 cells (Rankin et al., 1996). Th2 cytokines (IL-4 and IL- 13) drove IgE production in B cells, which mainly directed the further release of Th2-type cytokines in mast cells (Lee et al., 2015). In the present study, Bru-34 treatments also prevented serum OVA-specific IgE and IgG1 production significantly in allergic mice. Furthermore, our data also showed that Bru-34 significantly inhibited the secretion of IL-4 in IgE-activated mast cells. Taken together, the results suggested that Bru-34 had a potential of anti-airway inflammation associated with the regulation of the Th1/Th2 balance.

Although the specific cellular level mechanisms of asthma are not precisely defined, asthma is currently characterized as a chronic airway inflammatory disorder associated with the presence of activated Th2 lymphocytes, eosinophils and basophils (Ci et al., 2011). The IgE activated mast cells and basophil degranulations and release of mediators of allergic inflammation are the key events in the pathogenesis of allergic asthma (Gould and Sutton, 2008). The activated Syk plays a critical role in IgE-induced FcεRⅠ-dependent allergic inflammation, activating multiple inflammatory downstream signaling pathways, including the nuclear factor-κB (NF-κB) pathway, phospholipase A2 (PLA2) and phospholipase Cγ1 (PLCγ1)- mediated calcium influx, which regulate pro-inflammatory cytokine gene expression, such as TNF, IL-1β, et al (Ulanova et al., 2005; Gilfillan and Rivera, 2009; Lu et al., 2014).

Based on the previous work, in this study, we further investigated potential mechanisms whereby Bru-34 regulated the airway inflammation, by focusing on the activation of Syk signaling. The results showed that the expression and phosphorylation of Syk were significantly suppressed in both *in vivo* and *in vitro* allergic inflammatory models with Bru-34 treatments. Furthermore, we also found that Bru-34 could directly inhibits Syk kinase activities *in vitro*. Meanwhile, Bru-34 also down-regulated the expression of *p*-cPLA2, NF-κB and p-NF-κB, downdream of syk signaling. Interestingly, our results also showed that there was a close association between the Bru-34 regulation of inflammatory mediators (TNF-α and IL-4) and activation of Syk, cPLA2 and NF-κB by using specific inhibitors. Furthermore, the expression of cPLA2 and NF-κB were shown to be superimposed inhibited by co-treating Bru-34 with cPLA2 or NF-κB inhibitors. The cPLA2 inhibitor significantly blocked the phosphorylation of NF-κB, while NF-κB inhibitor did not affect the protein expression and phosphorylation of cPLA2. Overall, these results suggested that Syk was an important target, through which Bru-34 suppressed the inflammatory response, and cPLA2 and NF-κB also played important roles in the coordinated inflammatory response.

In conclusion, the present studies revealed that Bru-34 could modulate the allergic airway inflammation caused by Th1/Th2 imbalance, and its molecular mechanism, at least partially, might be on the regulation of Syk-cPLA2-NF-κB pathway.

## Data Availability

The datasets generated for this study are available on request to the corresponding author.
